# The wellbeing needs of social housing tenants in Australia: an exploratory study

**DOI:** 10.1186/s12889-022-12977-5

**Published:** 2022-03-24

**Authors:** Megan Freund, Robert Sanson-Fisher, David Adamson, Grace Norton, Breanne Hobden, Matthew Clapham

**Affiliations:** 1 Health Behaviour Research Collaborative, School of Medicine and Public Health, Faculty of Health and Medicine, University of Newcastle, NSW Callaghan, Australia; 2 Priority Research Centre for Health Behaviour, Faculty of Health and Medicine, The University of Newcastle, Callaghan, NSW 2308 Australia; 3 Hunter Medical Research Institute, New Lambton, NSW Australia; 4 Compass Housing Services Co Ltd, Hamilton, NSW Australia; 5 University of South Wales, Cardiff, South Wales UK; 6 Clinical Research Design and Statistics Support Unit, Hunter Medical Research Institute, New Lambton Heights, NSW Australia

**Keywords:** Social housing, Public housing, Tenants, Wellbeing, Vulnerability, Unmet needs, Aboriginal and Torres Strait Islander

## Abstract

**Background:**

Social housing provides homes for some of the most vulnerable in society. Those in social housing often have complex issues that may require support. Limited research has examined the unmet needs of those living in social housing from the tenant perspective. This exploratory study aimed to embark on filling this gap.

**Methods:**

A cross-sectional study survey of adult social housing tenants in New South Wales, Australia. Consenting tenants completed a survey asking about their support needs across five domains: transport, employment and financial stress; housing and safety; health and health behaviour; access to services; and control over one’s life. Negative binomial regression analysis was used to examine associations between the mean number of support needs and characteristics.

**Results:**

Of the 104 tenants invited, 101 agreed to participate (97%) of which 100 completed the survey. Paying unexpected bills’ (43%), feeling sad or anxious (40%), feelings of anger or frustration (34%), and memory or concentration problems (33%) were the most prevalent reported needs. Other needs included antisocial behaviour from neighbours (31%) and having control in the direction your life is taking (27%). Seventy-five percent of tenants reported at least one need, with an average of seven needs across the sample (median 5.5, range 0–24). Tenants who identified as Aboriginal or Torres Strait Islander had a higher number of needs compared to other tenants (RR 1.87 95% CI 1.08 to 3.35).

**Conclusions:**

More research describing tenant wellbeing needs is required to guide initiatives improving tenant wellbeing. Development of a standardised tool to measure and prioritise tenant wellbeing needs would be beneficial. There is a need for well-controlled research to establish the potential effectiveness and cost effectiveness of initiatives implemented at the policy or housing provider level. Future research must consider the multifaceted needs of this population. Further, Aboriginal and Torres Strait Islander people are generally overrepresented in social housing in Australia, and in this study Aboriginal and Torres Strait Islander tenants reported a higher number of needs compared to non-Aboriginal and Torres Strait Islander tenants. Given this, future research should ensure measurement tools or strategy initiatives are culturally sensitive and appropriate, and are developed in collaboration with Aboriginal and Torres Strait Islander communities.

**Supplementary Information:**

The online version contains supplementary material available at 10.1186/s12889-022-12977-5.

## Introduction

Social housing aims to provide access to safe, secure and affordable housing for those with little to no opportunity for other suitable housing [1, 2]. In recent years in Australia and elsewhere, social housing has undergone a ‘residualisation’ process where housing has been increasingly allocated to those with complex issues [3–5]. This includes those with physical and psychosocial disability, who are at risk of homelessness, or are on very low incomes [6, 7]. In practice, this means that having a very low income alone is rarely enough to access social housing. This has created an ‘ambulance service’ provision addressing housing of the most vulnerable in society [4, 5].

The concept of vulnerability is prominent in the narratives of social inequity and is increasingly used to prioritise service provision [8]. An individual’s vulnerability is a combination of exposure to a crises or stress, capacity to cope with those stressors, and the severity of impact of those stressors on an individual’s life [9]. Social housing tenants can experience multiple stressors including mental health issues, unemployment, substance misuse or gambling issues, cognitive impairment and experience of domestic violence [10, 11]. This can have a significant impact to their wellbeing and ability to sustain their tenancy [12]. Although the core responsibilities of social housing providers is the management of property and tenancy, addressing the needs of tenants “beyond housing” is an increasingly essential and resource-intensive part of social housing providers’ remit [13].

To increase the likelihood of successful initiatives aiming to improve tenant wellbeing, there is a need to understand for which stressors tenants perceive support is needed and welcomed. An individual’s perspective of a need for help is important as it helps to avoid the ‘paternalistic labelling’ of those categorised as belonging to a vulnerable group. People positioned as vulnerable and experiencing significant adversity often display considerable strength in managing situations [14]. Previous research that has examined tenant perceptions of their wellbeing needs have predominantly used qualitative techniques [15–18]. For example, an Australian study reported that social housing tenants (*n*= 76) required more consistent support for a range of needs to enable them to maintain their tenancy [19]. These included financial management, employment and education, and practical help in building life skills and navigating systems. Tenants reported the need for social housing providers and authorities to be more responsive to disruptive tenants with a preference for support being provided to those tenants. Tenants also expressed the need for continuity in the person/s providing support so that relationships can be built, and a need to be treated with respect and empathy [19]. Similar themes were identified in a United States qualitative study involving tenants in social housing who had chronic diseases (*n*= 55). Tenants emphasised practical help, such as navigating the health care system and providing support on disease management, could be provided by the introduction of support workers [17].

Although the information gained from qualitative research provides rich data regarding the needs of social housing tenants, quantitative research enables an understanding of the most prevalent tenant stressors. Increasing prevalence data will help social housing providers and policy makers to target initiatives to improve tenant wellbeing. It will also help identify groups of tenants with multiple needs who may require more intense or sustained support. This study therefore aimed to examine, among a sample of adult social housing tenants the: i) prevalence of wellbeing issues that tenants perceived a need for support; ii) average number and range of issues for which tenants indicated needing support; iii) demographic characteristics associated with the overall number of issues where tenants indicated they needed support. The study also examined the acceptability of asking about tenants’ support needs.

## Materials and methods

### Design and setting

In Australia, social housing can be managed by state and territory housing authorities (public housing), community-based organisations (community housing) or by Aboriginal and Torres Strait Islander communities (Indigenous community housing). State and territory housing authorities also manage state owned and managed Aboriginal and Torres Strait Islander housing. This cross-sectional study explored the wellbeing needs of adult social housing tenants of one community housing provider located in New South Wales (NSW). The community housing provider is an international, not-for-profit organisation and manages social housing portfolios throughout NSW, including in regional and remote areas. The housing provider’s tenant profile is typical of Australian social housing tenants with tenants including lone males, single parents, the elderly and those with physical and mental health challenges. The housing provider employs staff who support tenants to sustain their tenancies and provide community development activities to promote social cohesion and reduce anti-social behaviour. These services are funded from the Commonwealth Rental Assistance that accrues to Community Housing providers and these ‘more than bricks and mortar’ activities are generally less available in public housing communities where this funding path does not exist. The study was approved by the University of Newcastle Human Research Ethic Committee (Approval no. H-2018-0153).

### Tenant eligibility

Tenants aged 18 years of age and older who were the primary tenant of the household were eligible to participate in the study. Primary tenant refers to the person whose name appears on the tenancy agreement. Primary tenants who were recognised by the housing provider to have serious anti-social behaviour were deemed ineligible to participate (e.g. activities that place the safety of a household members, neighbours or housing provider staff at risk).

### Recruitment and data collection

From April to June 2019, the housing provider’s Tenant Engagement Officers (TEOs) approached consecutive eligible tenants about their potential participation in the study during their usual scheduled home visits. TEOs are support staff with established encouraging relationships with each tenant, rather than the tenancy managers who provide the compliance role. They perform a community development role, implementing engagement and cohesion strategies. This creates a relatively informal and supporting relationship with tenants, distinct from the tenancy management role of rent and property officers. TEOs provided eligible tenants with an information letter and a verbal explanation of the study. Tenants were assured that the research was being undertaken by university researchers and neither the housing provider nor the staff member would have access to the information they provided. If the tenant agreed to participate, they completed a pen and paper survey (10–15 min’s duration) at that time. Sole primary tenants were asked about their own wellbeing needs. Primary tenants with other adult household members were asked about the needs across all adults in the household. Completion of the survey was considered as consent to participate. Participating tenants received a $15 grocery gift voucher to reimburse them for their time. Housing provider staff kept a log of consenting and non-consenting tenants.

### Measures

#### Tenant characteristics

The survey gathered general tenant characteristic information including gender, age, level of education, Aboriginal or Torres Strait Islander status, employment status, and household composition.

#### Perceived wellbeing needs

Items regarding tenant wellbeing needs in the last 3 months across five domains were included in the survey. The domains, items and response scale were developed following an extensive review of the literature regarding factors associated with wellbeing [20, 21] generally and for social housing tenants specifically [11, 16–18, 22], and in discussion with experts in the field and with social housing providers. The survey was pilot tested with 10 tenants and revised as required based on housing provider staff and tenant feedback.

Tenants were asked to indicate their need for help in the last three months for each listed item. The transport, employment and financial stress domain included six items (e.g. getting the right skills for a job, transport if there was an emergency, budgeting to make ends meet). For example, ‘In the last 3 months did you need help getting the right skills for a job?’. The housing and safety domain included nine items (e.g. overcrowding at home, antisocial behaviour from neighbours). The health and health behaviour domain included 11 items (e.g. feelings of sadness or anxiety, feeling part of the community, smoking), the access to services domain included eight items (e.g. accessing assistance services, dealing with legal issues), and control over one’s life domain included four items (e.g. having control over the suburb that you live in).

Response options for each item were ‘I had enough help’; ‘I could’ve used a little more help; ‘I could’ve used a lot more help’; or ‘I didn’t need help’. The type of response chosen for this study was designed to better understand if the tenants desired assistance with an issue rather than if the issue existed per se. It was determined this type of information would be more useful to policy makers and housing providers when planning and prioritising future initiatives to address tenant wellbeing needs.

#### Survey acceptability

Survey acceptability was measured by level of agreement (five-point Likert scale of strongly agree to strongly disagree) with three questions asking respondents whether they found: i) questions easy to understand; ii) they felt comfortable answering all the questions; and iii) the survey was too long.

### Statistical analysis

Descriptive statistics were used to present the characteristics and the wellbeing needs of tenants. For the wellbeing needs items, response options were collapsed into ‘I could have used help’ (‘I could’ve used a little more help’ or ‘I could’ve used a lot more help’) and ‘I did not need help’ (‘I had enough help’ or I didn’t need help’). This was done to help identify the degree of perceived needs rather than help being needed per se. A count of the number of items (a total of 38 items) participants indicated they ‘could have used help’ was calculated. The average number, median and range was also calculated.

Negative binomial regression analysis was undertaken to examine the association between the mean count of items where tenants indicated they could have used help and tenant characteristics. Results are presented as rate ratios (RR). Rate ratios represent a relative change in the expected count compared to a reference category or for continuous variables, the relative change in expected count for a 1-unit increase. Tenant characteristics included gender; age (continuous variable); level of education (did not complete high school/high school or higher); Aboriginal or Torres Strait Islander status (yes/no); employment status (unemployed or unable to work/other); if only adult in household (yes/no) and; if any children live in the household (yes/no). Crude and multivariable rate ratios (RR) with 95% confidence intervals (CI) along with p-values from likelihood ratio tests were provided. Characteristics identified at a *p*-value < 0.05 were considered statistically significant.

Upset plots were used to visualise the intersections of participant needs items and are provided as supplemental material [23]. The upset plots show the intersection of needs items where participants ‘could have used help’ within each domain, ordered by the largest.

## Results

One hundred and four primary tenants were invited to participate in the study and 101 tenants consented (97% consent rate). One participant did not provide any data regarding unmet needs and was excluded from the sample. As such, the final sample size for analysis was 100 tenants.

### Tenant characteristics

Just over half of participants were the only household member (51%). Majority of were female (63%) and were aged 45 years or over (62%). Twenty-one percent of the participants identified as being Aboriginal or Torres Strait Islander (see Table 1). The characteristics of the participating sample were generally comparable with community housing provider’s tenant profile although females and older people were overrepresented in the study compared to the housing provider tenant population. The participant characteristics were comparable with those reported for Australian social housing tenants generally (62% female, 55% single adult households, and 73% aged 45 years or over) [24].

**Table 1:** **Sample characteristics (*N* = 100)**^a^

Characteristic	n (%)
Gender	
Female	62 (63)
Age	
18–24	7 (8)
25–34	11 (12)
35–44	17 (19)
45–54	20 (22)
55–64	19 (21)
65–74	14 (15)
75 +	4 (4)
Highest completed education level	
Primary school	1 (1)
Some high school	25 (25)
Completed Year 10	28 (28)
Completed high school	8 (8)
TAFE certificate or diploma	32 (32)
University degree or higher	5 (5)
Current employment status	
Full time work	0 (0)
Part time/ casual work	10 (10)
Home duties	9 (9)
Unemployed	33 (34)
Unable to work for health reasons	27 (28)
Retired	18 (18)
Student	1 (1)
Aboriginal or Torres Strait Islander	21(21)
Housing composition	
Lives alone	50 (51)
Other adults in household	17 (17)
Children in household	17 (17)
Other adults and children in household	14 (14)

^a^Sample size varies per characteristic (ranges 92 – 100) due to missing data

### Perceived wellbeing needs

The highest identified needs were related to ‘paying unexpected bills’ (43%), ‘feeling sad or anxious’ (40%) and ‘feeling angry or frustrated’ (34%). Table 2 displays the tenant needs in ranked order. Of the participating tenants, 75% identified at least one need. The mean number of needs across all participants was seven, with a median of 5.5 and ranging from 0–24. The upset plot analyses generally found that largest group in each domain was not needing help on all items. Detailed results from the upset plot analyses are provided in Supplementary file 2.

**Table 2:** Prevalence of tenant wellbeing needs in the last three months by domain (*N* = 100)

Need	Could have used help n (%)
Paying unexpected bills (e.g. broken fridge)^1^	42 (43)
Feelings of sadness or anxiety^3^	39 (40)
Feelings of anger or frustration^3^	34 (34)
Memory or concentration problems^3^	33 (33)
Worrying about the future^3^	32 (32)
Antisocial behaviour from neighbours^2^	30 (31)
Budgeting to make ends meet^1^	30 (30)
Finding a job^1^	28 (29)
Getting the right skills for a job (e.g. training)^1^	27 (28)
Control over the direction of your life is taking^5^	27 (27)
Safety in your housing block^2^	24 (24)
Safety in your neighbourhood^2^	24 (24)
Noise from surrounding homes^2^	23 (23)
Finding someone to talk to about your day-to-day problems^3^	23 (23)
Feeling part of the wider local community^3^	22 (22)
Your neighbourhood being dirty or run down^2^	22 (22)
Accessing aged care services *(if* ≥ *65yrs or more, n* = *18)*^4^	4 (22)
Transport if there was an emergency^1^	19 (19)
Accessing assistance services (e.g. Salvation Army)^4^	17 (17)
Being able to rent privately (i.e. move out of social housing)^5^	16 (17)
Smoking^3^	16 (16)
Dealing with social security payment provider (i.e. Centrelink)^4^	16 (16)
Dealing with your community housing provider^4^	15 (15)
Vandalism or damage to your property^2^	15 (15)
Control over the type of house/unit you live in (e.g. no. bedrooms)^5^	14 (14)
Transport to appointments (e.g. to the doctor)^1^	13 (13)
Dealing with National Disability Insurance Scheme^4^	13 (13)
Legal issues^4^	12 (12)
Dealing with police^4^	12 (12)
Dealing with the justice system^4^	11 (11)
Having control over the suburb that you live in^5^	11 (11)
Discrimination/racism^2^	9 (9)
Day to day activities (e.g. washing or dressing)^3^	9 (9)
Alcohol problems^3^	6 (6)
Gambling problems^3^	5 (5)
Drug problems^3^	4 (4)
Overcrowding at home^2^	4 (4)
Violence in your household^2^	2 (2)

***Note*:** Superscript numbers indicate domain category

^1^Transport, employment and financial stress

^2^Housing & Safety

^3^Health and wellbeing

^4^Access to services

^5^Life control

### Association between tenant descriptors and number of unmet needs

Tenants who identified as Aboriginal or Torres Strait Islander reported, on average, a higher number of needs compared to those who did not identify as Aboriginal or Torres Strait Islander (RR 1.87 95% CI 1.08 to 3.35). No other tenant characteristics were associated with the number of needs (see Table 3).

**Table 3:** Assocation between the mean number of needs and tenant characteristics

**Category**	**Univariate**			**Multivariate**	
	**Mean (SD)**	**RR**^**a**^** (95%CI)**	***P***	**RR**^**b**^** (95%CI)**	***P***
**Age**		0.97 (0.96–0.99)	0.002	0.99 (0.97–1.02)	0.538
**Gender**					
Male	8.0 (6.2)		0.287		0.096
Female	6.1 (6.3)	0.77 (0.46–1.25)		0.62 (0.35–1.09)	
**Aboriginal/Torres Strait Islander**					
No	5.9 (5.9)		0.013		0.026
Yes	11.5 (6.8)	1.96 (1.15–3.51)		1.87 (1.08–3.35)	
**Employment**					
Unemployed	8.2 (6.6)		0.015		0.275
Unable to work (health)	8.2 (6.5)	1.00 (0.57–1.76)		1.05 (0.54–2.07)	
Retired	2.8 (3.7)	0.34 (0.17–0.67)		0.52 (0.23–1.21)	
Other	8.0 (7.0)	0.97 (0.53–1.83)		0.81 (0.42–1.59)	
**Education**					
Year 12/TAFE/Higher	8.2 (6.6)		0.385		0.520
Year 10	6.5 (6.6)	0.79 (0.45–1.42)		0.80 (0.48–1.35)	
Primary/some high school	5.5 (6.0)	0.67 (0.38–1.22)		0.72 (0.39–1.36)	
**Other adults**					
No	8.0 (6.6)		0.141		0.239
Yes	5.5 (6.0)	0.68 (0.42–1.14)		0.74 (0.45–1.23)	
**Children**					
No	6.6 (6.3)		0.279		0.387
Yes	8.6 (6.8)	1.31 (0.81–2.16)		1.35 (0.68–2.64)	

^a^ *RR*  Rate Ratios

^b^ *CI*  Confidence Interval

### Acceptability

Ninety percent of participants indicated the survey questions were easy to understand, 90% indicated they felt comfortable answering all questions, and 18% of respondents indicated the survey was too long.

## Discussion

This is one of the first studies to quantitatively examine perceived support needs among tenants living in social housing within the Australian context. This study found paying unexpected bills was selected by the highest proportion of tenants. Given that very low income is a typically a criterion for accessing social housing, it is not unexpected that financial stress is a primary concern among tenants. Previous research has indicated a correlation between financial stress and health and wellbeing for social housing tenants [25]. Four out of five of the highest reported needs were related to mental wellbeing, which included feelings of sadness and anger, concentration problems and concern about the future. In line with previous research [19], several practical needs were also identified by a high proportion of tenants (e.g., help with budgeting to make ends meet and finding a job).

Tenants indicated that they needed support across a multitude of issues (an average of seven concurrent needs per tenant). This is not surprising given the transition from a social housing system designed to house the blue-collar workforce of the 1950-1970s [26], to being a resource allocated to only those with the greatest need [3, 4, 27]. The multiple, concurrent and interrelated needs (e.g. financial stress causing anxiety) found in the current study intuitively suggests the need for a co-ordinated wraparound approach [12]. This has in part been reflected in recent policy changes in a NSW program that has funded community housing organisations to develop new housing stock, with tenants encouraged to participate in a ‘Tailored Coordinated Support’ program that meets their support needs [28]. The NSW Future Directions housing policy also identifies an aspiration that 5% of tenants will improve their social and employment outcomes and transition to the private sector rental market. Recently, the COVID related response to homelessness in NSW, the Together Home program, has provided funding for additional support services for those housed during the pandemic. The multiple needs of vulnerable families are also addressed by such as the Healthy Homes and Neighbourhoods initiative in inner Sydney, NSW [29].

In line with consumer-directed care in public and social services, such a wraparound approach should encourage tenants to take an active role in decisions about their wellbeing, assist tenants to understand their options for support, while helping them to understand and apply their values and preferences in making decisions [30]. Whilst the value of a wraparound support approach has been recognised internationally in the Housing First response to homelessness [31], it has not been robustly examined in relation to the general social housing population. This highlights the need for further research to inform multi-component and tailored intervention strategies aimed at improving tenant wellbeing in the social housing system.

Twenty-one percent of tenants identified as being Aboriginal or Torres Strait Islander compared to 3.3% for the overall population in the same region in which the study was undertaken [32]. This finding is line with national data that reports an overrepresentation of Aboriginal and Torres Strait Islander people in social housing [33]. Around 13% (53,700) of all social housing households in Australia include an Aboriginal and Torres Strait Islander member and, 21% of Aboriginal and Torres Strait Islander households reside in social housing [24]. Tenants’ who identified as Aboriginal or Torres Strait Islander reported a higher number of needs compared to non-Aboriginal and Torres Strait Islander people. These findings align with the well documented social and wellbeing disparities between Aboriginal and Torres Strait Islander and non-Aboriginal and Torres Strait Islander Australians [34], and highlights the continued impact of historical events such as colonialization, the stolen generation and intergenerational trauma [35]. Culturally sensitive and strengths-based programs, which are formed in collaboration with Aboriginal and Torres Strait Islander communities, is needed to support the wellbeing of Aboriginal and Torres Strait Islander social housing tenants.

Interestingly, a number of issues were not identified as an area requiring support by many social housing tenants (e.g. dealing with the justice system or police, alcohol or other drug use, and violence in the home). There may be several potential explanations for this finding. First, although social housing is often stereotyped as a hotbed of crime [36], such typecasting is unlikely to be representative of the majority of tenants. Second, social desirability bias may have contributed to the relatively small proportion of tenants reporting needing help with these issues. Third, criminal behaviour can ultimately lead to eviction for social housing tenants [37], and this may have exacerbated tenant reluctance to report a need for help for these behaviours. Last, although tenants may experience these issues, tenants may perceive that they did not require support for this issue (e.g. did not perceive it to be a problem or simply did not want any assistance with the issue).

### Limitations and strengths

This study used a relatively small non-random sample of social housing tenants of one social housing provider in NSW, hence the outcomes reported may not be generalizable to the broader social housing tenant population. The sample size may also have impacted the ability for finding other significant associations in the regression analysis. As housing provider staff were present while tenants completed the survey, tenants may have been concerned about the confidentiality of their answers and this may have resulted in social desirability bias. Whilst not fully removing the landlord/tenant power relationship, they were able to underline the voluntary and anonymous character of the survey and stress the independent administration of the process and analysis of the results by the University Team. The survey tool was not validated and so the outcomes may be affected by measurement error. For multi-adult households, the primary tenant provided a third-party report of needs for all adults in the household which may have resulted in under- or over-reporting of need for help. The study may not have included all indicators that may affect tenant wellbeing (e.g. if reside in a housing block or a stand-alone house). This study did obtain a high consent rate and the majority of respondents found the survey to be acceptable. This points to the feasibility of future research examining tenant wellbeing needs on a wider scale.

## Conclusions

The experience of tenants in social housing has, in recent years been conceptualised as a housing’pathway’ that promotes transition of tenants to the private rental sector [19, 38]. However, with allocations almost entirely based on ‘greatest needs’, the prospect of transition is limited for the majority of social housing tenants. In public housing, 43% of tenants have been in the tenancy for over ten years [39]. This suggests the desirability of long-term support partnerships between the tenant, the social landlord and appropriate support agencies. The multiple needs for tenants found by this study also indicates a need for multicomponent programs to provide adequate support. This level of support will often be critical for the maintenance of the tenancy [12]. Given this was an exploratory study, more research that describes the prevalence and types of tenant wellbeing needs is required to provide guidance to social housing providers and policy makers when developing initiatives to improve tenant wellbeing. The development of a standardised tool to measure and prioritise tenant wellbeing needs, similar to that developed for prioritising housing support for the homeless [40, 41], would be a useful next step. Additionally, there is a need for well-controlled research to establish the potential effectiveness and cost effectiveness of initiatives implemented at the policy or housing provider level. Any future research in this area must take account of the multifaceted needs of this population group and ensure the research’s relevance and applicability to Aboriginal and or Torres Strait Islander people.

## Supplementary Information

**Additional file 1.**

**Additional file 2.**

## Data Availability

The datasets generated and/or analysed during the current study are not publicly available as ethical approval states the data is to be stored on University of Newcastle secured files. The data may be available from the corresponding author on reasonable request.
