# Supplementary material for: The wellbeing needs of social housing tenants in Australia: an exploratory study
Source: BMC Public Health. 2022 Mar 24;22:582. doi: 10.1186/s12889-022-12977-5 (PMC8953361; doi:10.1186/s12889-022-12977-5)
Supplement: Supplementary file 1 — Additional file 1. [file 12889_2022_12977_MOESM1_ESM.docx]

**Section 1: About you and your household**

1. **How many other adults currently live with you in the household? These are people aged ≥18 years of age who live with you and share common household arrangements**

- None, I am the only adult ***OR***
- Enter no. ______

1. **How many children live with you in the household at least half of the time?**

- None ***OR***
- Enter no. ______

**Section 2: Need for help**

The following questions ask about your need for help in last month. They include questions about things that might impact on your quality of life. For each item, please tick the response that best describes your need for help **in the last 3 months**. People have different experiences, so not all of the items will be relevant to you and your situation. In these cases please tick “I didn’t need help”.

Part 1. The following items relate to transport, employment and financial stress.

| **I didn’t need help** | | **I had enough**  **help** | **I could have used a little more help** | **I could have used a lot more help** | **I didn’t need help** |
| --- | --- | --- | --- | --- | --- |
|  | Transport to appointments (e.g. to the doctor) | _0_□ | _1_□ | _2_□ | _3_□ |
|  | Transport if there was an emergency | _0_□ | _1_□ | _2_□ | _3_□ |
|  | Getting the right skills for a job (e.g. training) | _0_□ | _1_□ | _2_□ | _3_□ |
|  | Finding a job | _0_□ | _1_□ | _2_□ | _3_□ |
|  | Paying unexpected bills (e.g. broken fridge) | _0_□ | _1_□ | _2_□ | _3_□ |
|  | Budgeting to make ends meet | _0_□ | _1_□ | _2_□ | _3_□ |

Part 2. The following items relate to housing and safety

| **In the last 3 months**  **did you need help with:** | | **I had enough**  **help** | **I could have used a little more help** | **I could have used a lot more help** | **I didn’t need help** |  |
| --- | --- | --- | --- | --- | --- | --- |
|  | Noise from surrounding homes | _0_□ | _1_□ | _2_□ | _3_□ |  |
|  | Your neighbourhood being dirty or run down | _0_□ | _1_□ | _2_□ | _3_□ |  |
|  | Antisocial behaviour from neighbours | _0_□ | _1_□ | _2_□ | _3_□ |  |
|  | Overcrowding at home | _0_□ | _1_□ | _2_□ | _3_□ |  |
|  | Discrimination/racism | _0_□ | _1_□ | _2_□ | _3_□ |  |
|  | Safety in your housing block? | _0_□ | _1_□ | _2_□ | _3_□ | |
|  | Safety in your neighbourhood? | _0_□ | _1_□ | _2_□ | _3_□ | |
|  | Vandalism or damage to your property | _0_□ | _1_□ | _2_□ | _3_□ | |
|  | Violence in your household | _0_□ | _1_□ | _2_□ | _3_□ | |

Part 3. The following items relate to your health and wellbeing

| **In the last 3 months**  **did you need help with:** | | **I had enough**  **help** | **I could have used a little more help** | **I could have used a lot more help** | **I didn’t need help** |
| --- | --- | --- | --- | --- | --- |
|  | Day to day activities (e.g. washing or dressing) | _0_□ | _1_□ | _2_□ | _3_□ |
|  | Alcohol problems | _0_□ | _1_□ | _2_□ | _3_□ |
|  | Drug problems | _0_□ | _1_□ | _2_□ | _3_□ |
|  | Smoking | _0_□ | _1_□ | _2_□ | _3_□ |
|  | Gambling problems | _0_□ | _1_□ | _2_□ | _3_□ |
|  | Memory or concentration problems | _0_□ | _1_□ | _2_□ | _3_□ |
|  | Worrying about the future | _0_□ | _1_□ | _2_□ | _3_□ |
|  | Feeling sad or anxious | _0_□ | _1_□ | _2_□ | _3_□ |
|  | Feeling angry or frustrated | _0_□ | _1_□ | _2_□ | _3_□ |
|  | Having someone to talk to about your day-to-day problems | _0_□ | _1_□ | _2_□ | _3_□ |
|  | Feeling part of the wider local community | _0_□ | _1_□ | _2_□ | _3_□ |

Part 4. The following items relate to access to services

| **In the last 3 months**  **did you need help with:** | | **I had enough**  **help** | **I could have used a little more help** | **I could have used a lot more help** | **I didn’t need help** |
| --- | --- | --- | --- | --- | --- |
|  | Dealing with Centrelink | _0_□ | _1_□ | _2_□ | _3_□ |
|  | Legal issues | _0_□ | _1_□ | _2_□ | _3_□ |
|  | Dealing with police | _0_□ | _1_□ | _2_□ | _3_□ |
|  | Dealing with the justice system | _0_□ | _1_□ | _2_□ | _3_□ |
|  | Dealing with Compass Services | _0_□ | _1_□ | _2_□ | _3_□ |
|  | Dealing with National Disability Insurance Scheme (NDIS)? | _0_□ | _1_□ | _2_□ | _3_□ |
|  | Access to Aged Care Services *(answer if 65yrs or more)* | _0_□ | _1_□ | _2_□ | _3_□ |
|  | Access to other services (e.g. Salvos) | _0_□ | _1_□ | _2_□ | _3_□ |

**Part 5**. The following items relate to the control of your life

| **In the last 3 months**  **did you need help with:** | | **I had enough**  **help** | **I could have used a little more help** | **I could have used a lot more help** | **I didn’t need help** |
| --- | --- | --- | --- | --- | --- |
|  | Having control over the type of house/unit you live in (e.g. number of bedrooms) | _0_□ | _1_□ | _2_□ | _3_□ |
|  | Having control over the suburb that you live in | _0_□ | _1_□ | _2_□ | _3_□ |
|  | Having control over the direction your life is taking | _0_□ | _1_□ | _2_□ | _3_□ |
|  | Being able to rent privately (i.e. move out of social housing) | _0_□ | _1_□ | _2_□ | _3_□ |

**SECTION 3: GENERAL QUESTIONS ABOUT YOU**

And now some general question about you.

1. **What is your gender?**

- Male
- Female
- Other

1. **What is your age?** _____
2. **What is the highest level of education that you have completed?**

- Never attended school
- Primary school
- Some high school
- Completed school certificate, Intermediate, Yr 10, 4th Form
- Completed HSC, Leaving, Year 12 or 6th Form
- TAFE certificate or diploma
- University, CAE, Degree or higher
- Other (please specify): ___________________

1. **Are you of Aboriginal and/or Torres Strait Islander origin?**

- No
- Yes, Aboriginal
- Yes, Torres Strait Islander
- Yes, both Aboriginal and Torres Strait Islander

1. **What best describes your current employment status?**

- Full time work
- Part time/ casual work
- Home duties
- Unemployed
- Unable to work for health reasons
- Retired
- Student

**SECTION 4: ACCEPTABILITY OF COLLECTING MBS/PBS DATA AND THE SURVEY**

Lastly, we would like to ask you what you thought about completing this survey.

| **When completing the survey, did you find….** | | **Strongly agree** | **Agree** | **Unsure** | **Disagree** | **Strongly disagree** |
| --- | --- | --- | --- | --- | --- | --- |
|  | The questions were easy to understand | _0_□ | _1_□ | _2_□ | _3_□ | _4_□ |
|  | You felt comfortable answering all the questions | _0_□ | _1_□ | _2_□ | _3_□ | _4_□ |
|  | The survey was too long | _0_□ | _1_□ | _2_□ | _3_□ | _4_□ |

**That’s the end of the survey.**

**Thank you taking the time to participate.**
