# Supplementary material for: The wellbeing needs of social housing tenants in Australia: an exploratory study
Source: BMC Public Health. 2022 Mar 24;22:582. doi: 10.1186/s12889-022-12977-5 (PMC8953361; doi:10.1186/s12889-022-12977-5)
Supplement: Supplementary file 2 — Additional file 2. [file 12889_2022_12977_MOESM2_ESM.docx]

Figures S1 to S5 present upset plots for the 5 domains of needs items. The bar height indicates the number of participants answering could have used help for the combination items indicated by black circles.

There were 45 participants who did not need any help for any item within the domain transport, employment and financial stress (First bar in Figure S1). 42 could have used help with “paying unexpected bills”. 27 could have used help with “Paying unexpected bills” and “Budgeting to make ends meet”. 16 could have used help with “Paying unexpected bills”, “Finding a job” and “Getting the right skills for a job”. 3 could have used help for all 6 items in the transport, employment and financial stress domain.

Inclusive intersection size

0

10

20

30

40


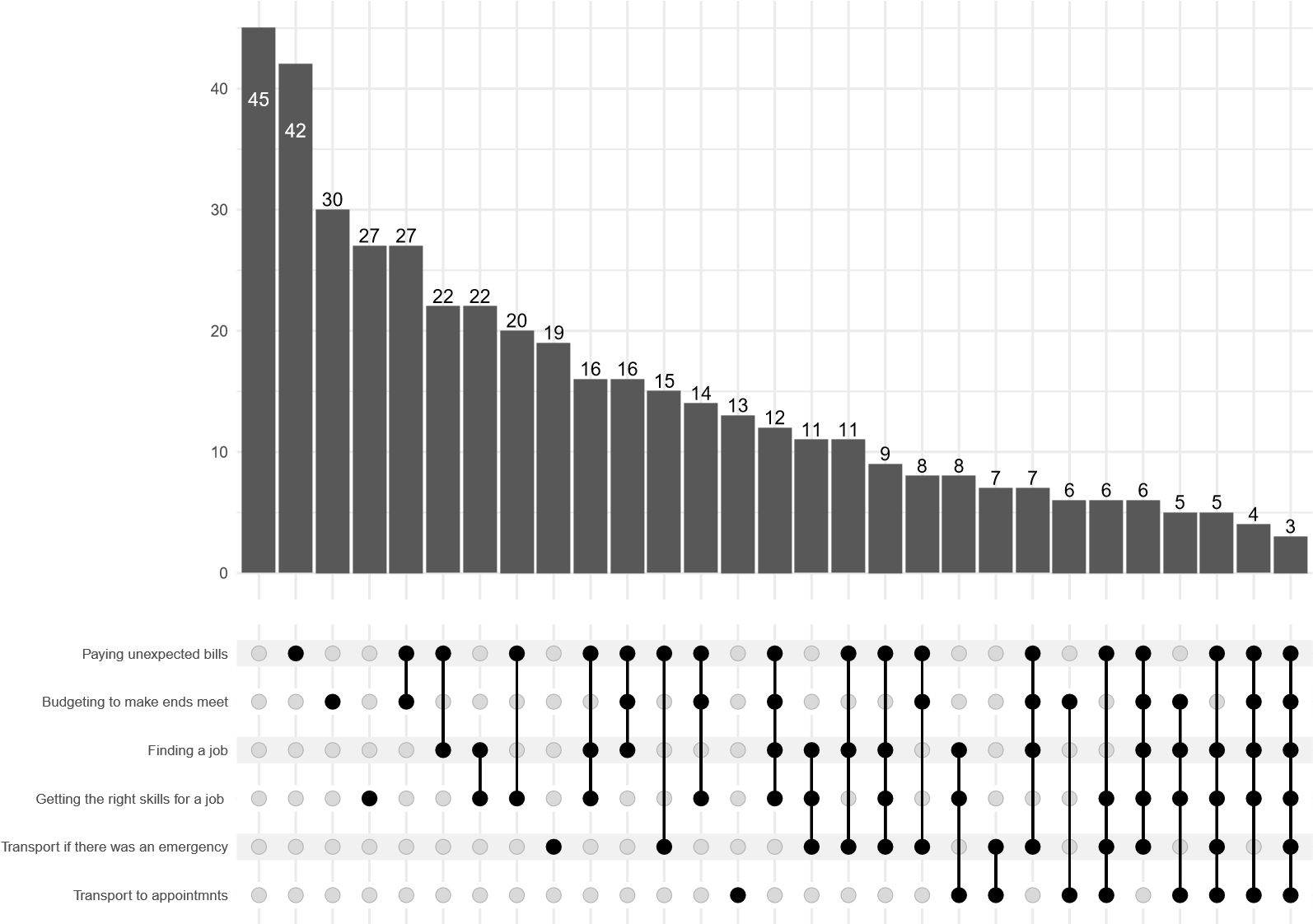


Set size Transport, employment and financial stress

Figure S1: Transport, employment and financial stress questions

There were 56 participants who did not need any help for any item within the domain housing and safety (First bar in Figure S2). 30 could have used help with “Antisocial behaviour from neighbours”. 19 could have used help with “Antisocial behaviour from neighbours” and “Noise from surrounding homes”. 12 could have used help with “Antisocial behaviour from neighbours”, “Safety in your neighbourhood”, “Safety in your housing block” and “Noise from surrounding homes”. No one could have used help for all 9 items in the Housing and safety.

Inclusive intersection size

0

10

20

30


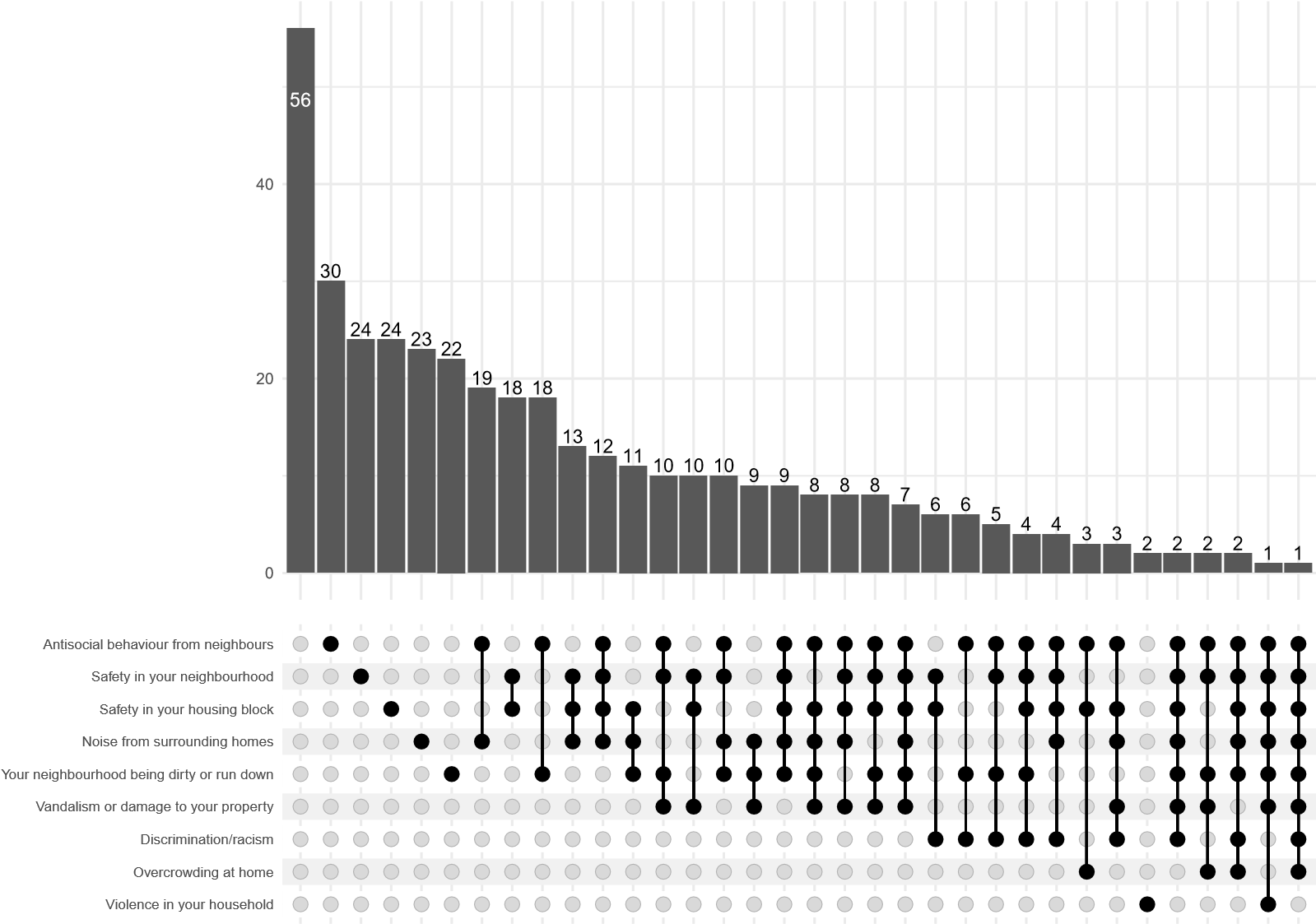


Set size Housing and safety

Figure S2: Housing and safety questions

There were 44 participants who did not need any help for any item within the domain health and wellbeing

(First bar in Figure S3). 39 could have used help with “Feelings of sadness or anxiety”. 27 could have used help with “Feelings of sadness or anxiety” and “Worrying about the future”. 20 could have used help with “Feelings of sadness or anxiety”, “Feelings of anger or frustration” and “Worrying about the future”.

Inclusive intersection size

0

10

20

30

40


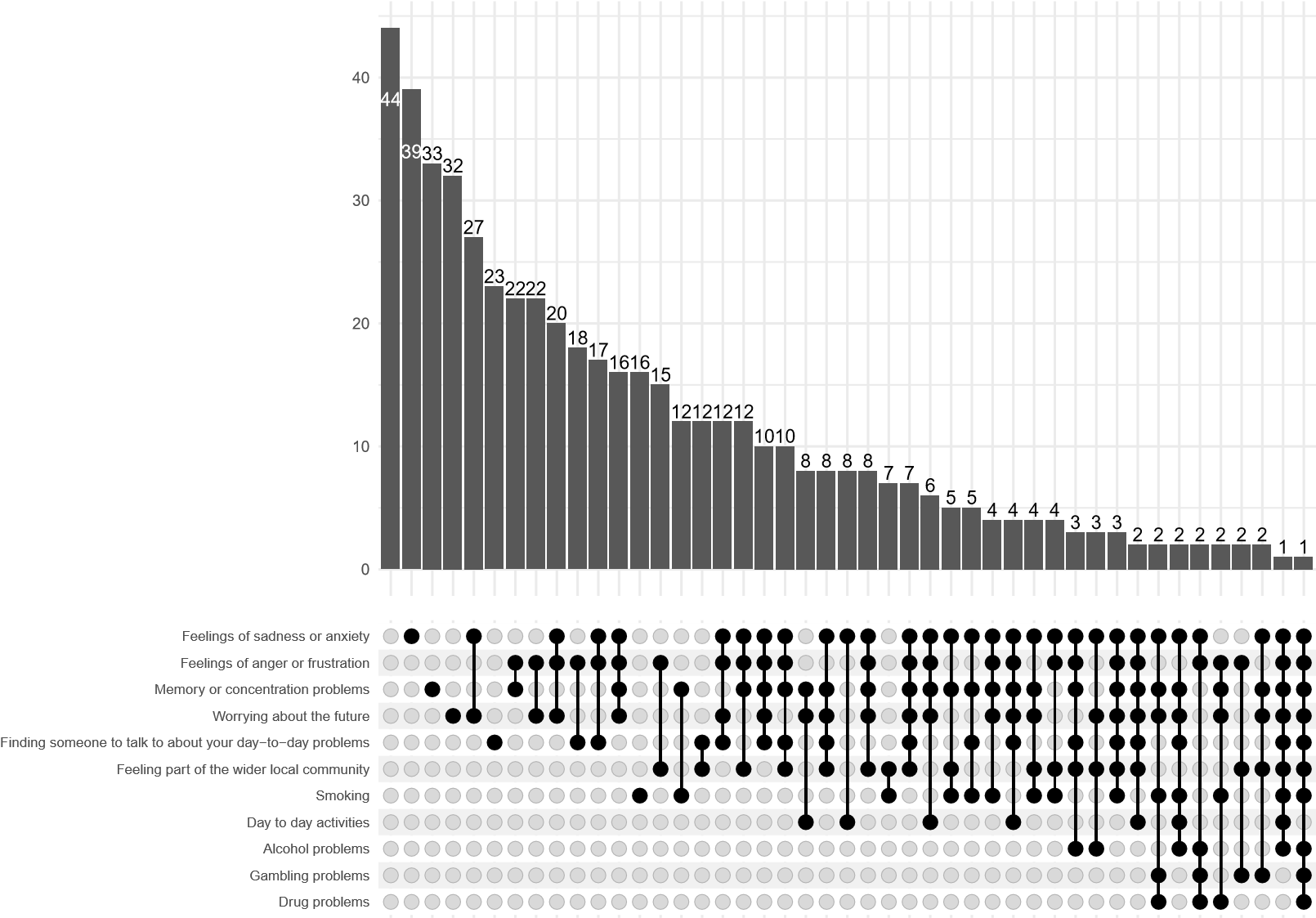


Set size Health and wellbeing

Figure S3: Health and wellbeing questions

There were 51 participants who did not need any help for any item within the domain access to services

(First bar in Figure S4). 13-17 could have used help with “accessing other services”, “Dealing with Centrelink”, “Dealing with Compass Services” or “Dealing with National Disability Insurance Scheme (NDIS)”. 6 participants could have used help with both “Accessing other services” and “Dealing with Centrelink”. 7 participants could have used help with “Dealing with police”, “Legal issues” and Dealing with the justice system”.

Inclusive intersection size

0

5

10

15


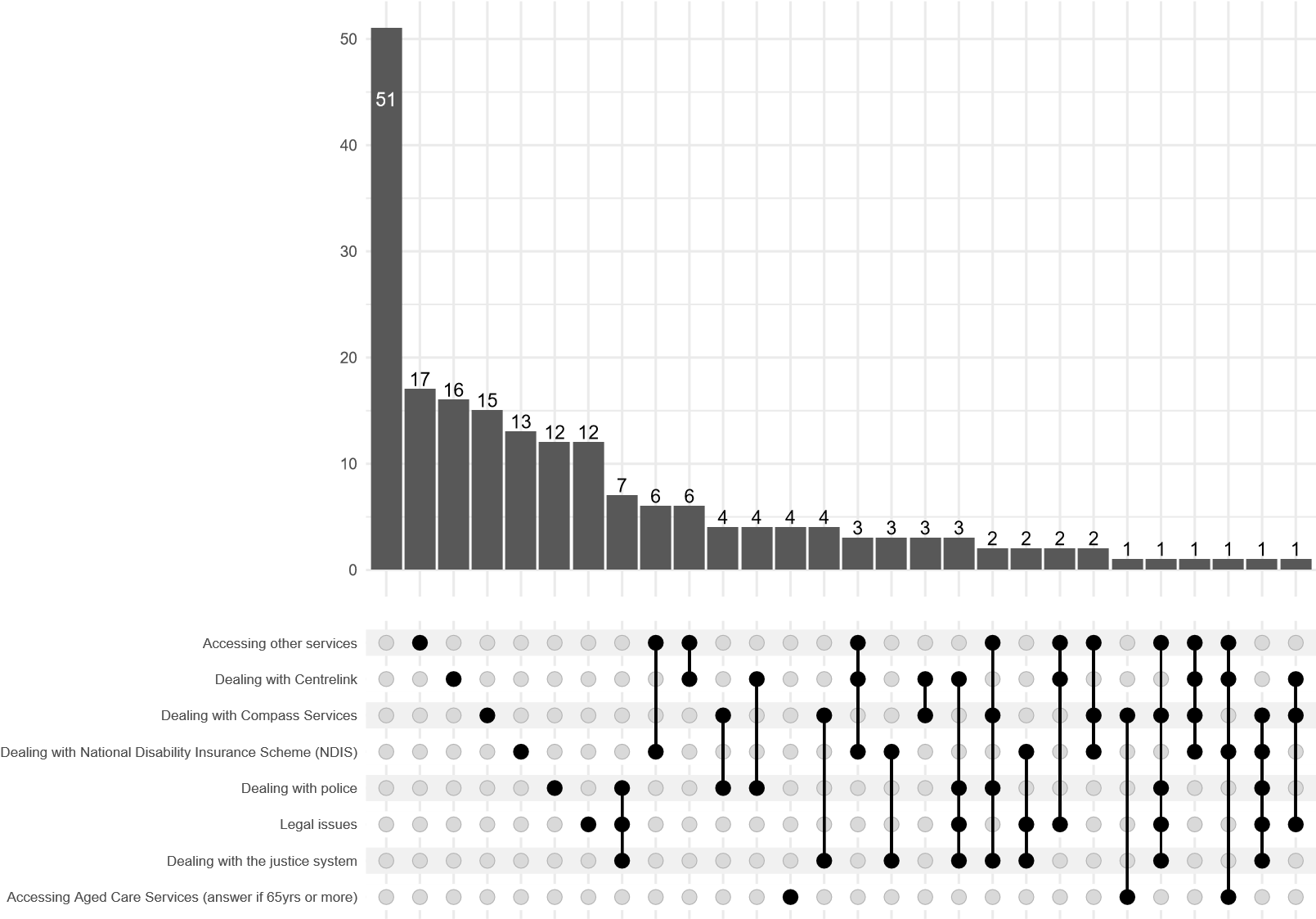


Set size Access to services

Figure S4: Access to services questions

There were 65 participants who did not need any help for any item within the domain life control (First bar in Figure S5). 27 could have used help with “Having control over the direction your lives are taking”. 5 could have used help for all 4 items in the life control domain.

Inclusive intersection size

0

10

20


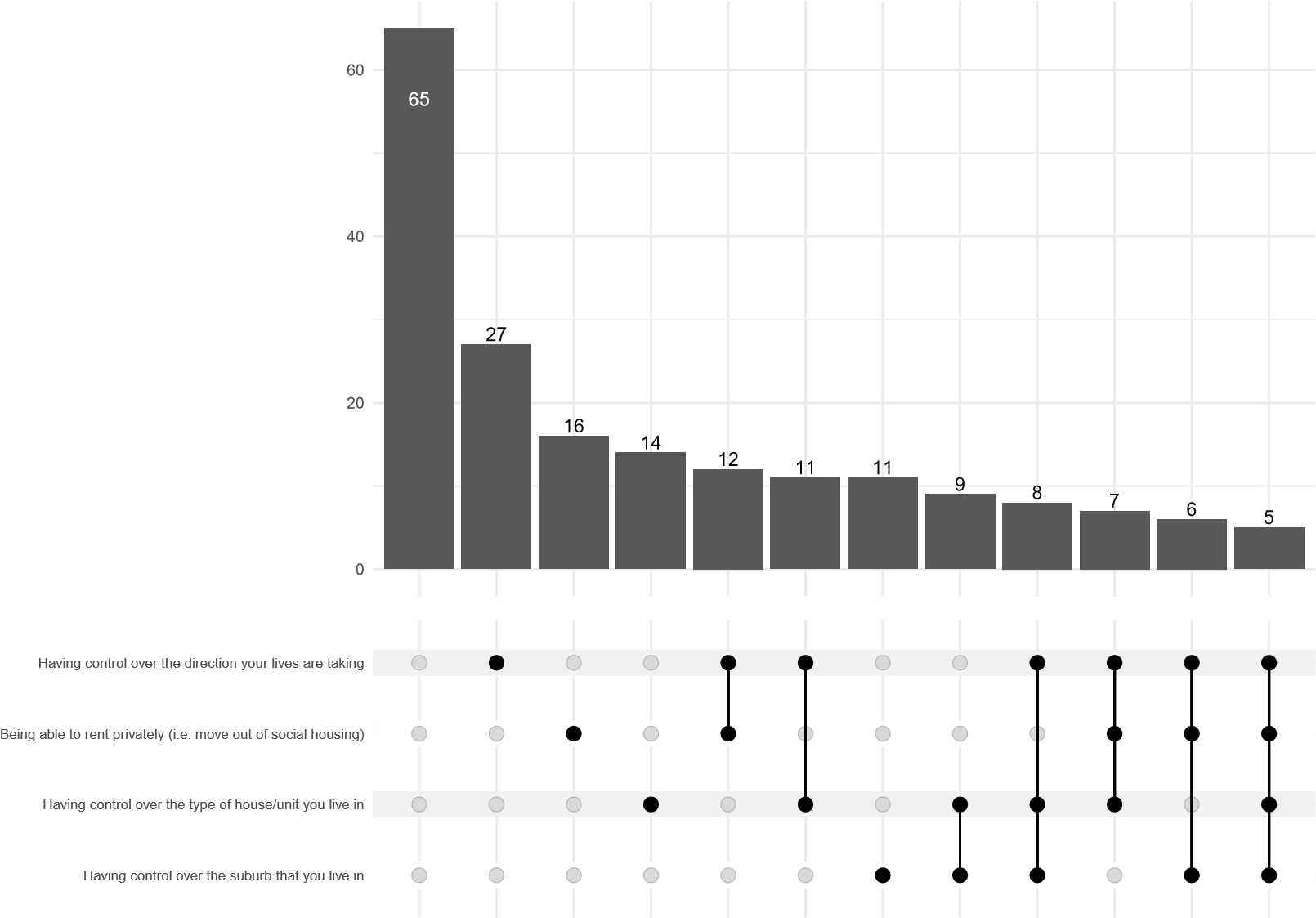


Set size Life control

Figure S5: Life control questions
